# Neurodegeneration or dysfunction in Phelan-McDermid syndrome? A multimodal approach with CSF and computational MRI

**DOI:** 10.1186/s13023-023-02863-7

**Published:** 2023-09-05

**Authors:** Sarah Jesse, Hans-Peter Müller, Hans-Jürgen Huppertz, Stephanie Andres, Albert C. Ludolph, Michael Schön, Tobias M. Boeckers, Jan Kassubek

**Affiliations:** 1 Department of Neurology, University Hospital Ulm, Oberer Eselsberg 45, D-89081 Ulm, Germany; 2 Swiss Epilepsy Clinic, Hospital Lengg, Zürich, Switzerland; 3 Medicover München Ost MVZ, Humangenetik, Munich, Germany; 4 German Centre of Neurodegenerative Diseases (DZNE), Ulm, Germany; 5 Institute of Anatomy and Cell Biology, Ulm University, Ulm, Germany

**Keywords:** Phelan-McDermid syndrome, *SHANK*3, Cerebrospinal fluid, Tau-protein, Amyloid-ß, Neurodegeneration, Dysfunction, Magnetic resonance imaging, White matter, DTI

## Abstract

**Background:**

Phelan-McDermid syndrome (PMS) is a rare multisystem disease with global developmental delay and autistic features. Genetically, the disease is based on a heterozygous deletion of chromosome 22q13.3 with involvement of at least part of the *SHANK3* gene or heterozygous pathogenic variants in *SHANK3*. Pathophysiologically, this syndrome has been regarded as a synaptopathy, but current data suggest an additional concept, since axonal functions of neurons are also impaired, thus, the specific pathophysiological processes in this disease are not yet fully understood. Since symptoms of the autism spectrum, regression, and stagnation in development occur, we investigated whether neuroinflammatory and neurodegenerative processes may also play a role. To this end, we analysed biomarkers in cerebrospinal fluid (CSF) and parameters from magnetic resonance imaging with high-resolution structural T1w volumetry and diffusion tensor imaging analysis in 19 Phelan-McDermid syndrome patients.

**Results:**

CSF showed no inflammation but abnormalities in tau protein and amyloid-ß concentrations, however, with no typical biomarker pattern as in Alzheimer’s disease. It could be demonstrated that these CSF changes were correlated with integrity losses of the fibres in the corticospinal tract as well as in the splenium and dorsal part of the cingulum. High CSF levels of tau protein were associated with loss of integrity of fibres in the corticospinal tract; lower levels of amyloid-ß were associated with decreasing integrity of fibre tracts of the splenium and posterior cingulate gyrus. Volumetric investigations showed global atrophy of the white matter, but not the grey matter, and particularly not in temporal or mesiotemporal regions, as is typical in later stages of Alzheimer’s disease.

**Conclusions:**

In summary, alterations of neurodegenerative CSF markers in PMS individuals could be demonstrated which were correlated with structural connectivity losses of the corticospinal tract, the splenium, and the dorsal part of the cingulum, which can also be associated with typical clinical symptoms in these patients. These findings might represent a state of dysfunctional processes with ongoing degenerative and regenerative processes or a kind of accelerated aging. This study should foster further clinical diagnostics like tau- and amyloid-PET imaging as well as novel scientific approaches especially in basic research for further mechanistic proof.

## Background

The Phelan-McDermid syndrome (PMS) is a disease (OMIM#606,232) characterized by a global developmental delay with motor deficits (muscle hypotonia), expressive and receptive language development delay, cognitive impairment, epilepsy, and other neuropsychiatric comorbidities such as mood disorders, regression, and autism spectrum disorders [1]. The clinical variability is wide, a finding that has not yet been conclusively explained pathophysiologically. The genetic changes underlying the syndrome also vary greatly from point mutations, deletions, inversions, translocations affecting in most cases *SHANK3* on chromosome 22q13.3 [2], i.e., SH3 domain and ankyrin repeat-containing protein. Currently, patients with a partial deletion in the chromosomal region 22q13 without *SHANK3* involvement have been recognized who have a similar clinical phenotype so that the nomenclature of the syndrome has recently been adjusted in PMS-*SHANK3* related or PMS-*SHANK3* unrelated [3].

*SHANK3* encodes structural proteins of the postsynapse of excitatory neurons [4]. The components of the postsynaptic density and in particular the SHANK proteins are presumably relevant for processes such as learning and long-term potentiation through the induction of plasticity via spine and synapse formation [5]. *SHANK3* is not only expressed within the brain in the hippocampus, amygdala, cingulate gyrus, and cerebellum [6], but also outside the brain in muscles [7] and neurons of the peripheral nervous system [8].

Meanwhile, there are also data that the PMS is not only a disease of the synapses, but also of the white matter with impairment of the directionality of certain fibre tracts [9, 10]. Thus, Phelan-McDermid syndrome as a multisystem disease has been well characterized in the genetic and clinical domains, but has pathophysiologically not yet been fully deciphered. For this reason, patients with the syndrome should receive extensive exclusion diagnostics of commonly reported comorbidities after receiving the genetically based diagnosis, especially since clinical organ involvement has been described as part of the disease [1].

The current study is based on multimodal data from diagnostic processes and addresses the following questions:

1. Can cerebrospinal fluid be used to demonstrate inflammatory or neurodegenerative processes in patients with PMS?
2. Is there a morphological correlate in the brain for changes in the CSF biomarkers that can be associated by means of advanced volumetric or microstructural MRI data?
3. Are there differences in the results related to the classification of patients into PMS-*SHANK3* related and PMS-*SHANK3* unrelated?

## Results

### CSF routine parameters

In the CSF routine parameters white cell count/µl, total protein in mg/dl, lactate in mmol/l and oligoclonal IgG, none of the patients showed any pathological findings. Results were displayed in a standard Reibergram and measured automatically in our hospital CSF laboratory according to established formulas of Prof. Reiber [11]. Here, we observed neither pleocytosis nor other signs of (auto-) inflammatory processes (Table 1).

**Table 1:** CSF parameters of PMS patients. Data including white cell count/µl, total protein in mg/dl, lactate in mmol/l, intrathecal IgG synthesis, oligoclonal bands, tau protein in pg/ml (reference range < 400 pg/mL), amyloid-ß in pg/ml (reference range > 600 pg/mL), CXCL13 in pg/ml, pNF-H in pg/ml, amyloid-ß 42/40, p-Tau 181 in pg/ml

No	White cell count	Total protein	Lactate	Intrathecal IgG synthesis	OCB	Tau	p-Tau	Aß	Aß 42/40	CXCl13	pNF-H
1	3	404	1,16	no	no	424	< 60	533	> 0,07	< 4	< 188
2	0	157	1	no	no	75	< 60	342	> 0,07	< 4	< 188
3	2	347	1,5	no	no	212	< 60	813	> 0,07	< 4	< 188
4	1	340	1,6	no	no	163	< 60	1088	> 0,07	< 4	< 188
5	0	549	1,85	no	no	452	< 60	1025	> 0,07	< 4	< 188
6	2	697	1,8	no	no	489	< 60	781	> 0,07	< 4	< 188
7	1	136	0,89	no	no	137	< 60	414	> 0,07	< 4	< 188
8	1	157	1,09	no	no	327	< 60	331	> 0,07	< 4	< 188
9	0	263	1,4	no	no	142	< 60	464	> 0,07	< 4	< 188
10	0	146	1,09	no	no	197	< 60	370	> 0,07	< 4	< 188
11	0	301	1,36	no	no	183	< 60	569	> 0,07	< 4	< 188
12	1	321	1,16	no	no	258	< 60	613	> 0,07	< 4	< 188
13	0	287	0,92	no	no	264	< 60	1137	> 0,07	< 4	< 188
14	1	171	0,98	no	no	462	< 60	803	> 0,07	< 4	< 188
15	1	256	0,92	no	no	357	< 60	1007	> 0,07	< 4	< 188
16	1	176	1,18	no	no	270	< 60	1195	> 0,07	< 4	< 188
17	3	140	1,17	no	no	81	< 60	332	> 0,07	< 4	< 188
18	0	137	0,96	no	no	544	< 60	618	> 0,07	< 4	< 188
19	1	185	1,08	no	no	201	< 60	663	> 0,07	< 4	< 188

Abbreviations: OCB = oligoclonal bands, Aß = amyloid-ß, CXCL13 = C-X-C motif chemokine 13, pNF-H = neurofilament heavy chain

### CSF inflammation and neurodegeneration markers

Normal values ​​were also shown for the cytokine CXCL13 as an inflammation marker (in pg/ml, cut-off < 4). In a next step, markers were examined that reflect neurodegeneration. For pNF-H (in pg/ml, cut-off < 188), none of the patients showed abnormal values ​​either. The degeneration markers tau protein (reference range < 400 pg/mL) and amyloid-ß (reference range > 600 pg/mL) revealed pathological values ​​for one of the two parameters in 63% of all patients (only tau protein in 26%, only amyloid-ß in 42%). It is noteworthy that only one patient had abnormal values ​​for both biomarkers. The amyloid-ß quotient 1–42/1–40 (reference range > 0.07) was not abnormal in any of the patients, p-Tau 181 (reference range < 60 pg/ml) also showed normal values ​​in all patients. Therefore, there seemed to be no typical Alzheimer’s constellation of these biomarkers in laboratory CSF tests (Table 1).

### Associations of CSF

Since neurodegenerative processes in PMS have not yet been described, the question arose whether the current results could be dependent on different variables. No associations were observed between the biomarkers tau protein / amyloid-ß and the variables age, gender, genetic findings (in particular, no differences with regard to *SHANK3* related or *SHANK3* unrelated), language, motor function, ASD, epilepsy, cognition, and ADHD, respectively (data not shown).

After the CSF data gave no indication that the typical biomarker constellation of Alzheimer’s disease was present and there was also no association with clinical/genetic data, the hypothesis was pursued that the biomarker changes of tau/amyloid-ß might be an expression of dysfunctional processes in our patients` brains. Therefore, cerebral morphology structures, separately for white and grey matter, were further investigated using DTI and atlas-based volumetry.

### Whole brain-based spatial statistics of DTI

The unbiased whole brain-based comparison at the group level demonstrated multiple clusters of regional FA decreases. The pattern of the microstructural alterations consisted of one interconnected cluster covering large WM areas in all lobes of the brain as well as in the brainstem, and in the cerebellum (> 500 000 m³, p < 0.000001), indicative of globally altered cerebral white matter microstructure.

### Associations of FA with the biomarkers tau protein and amyloid ß

**Fig. 1:**
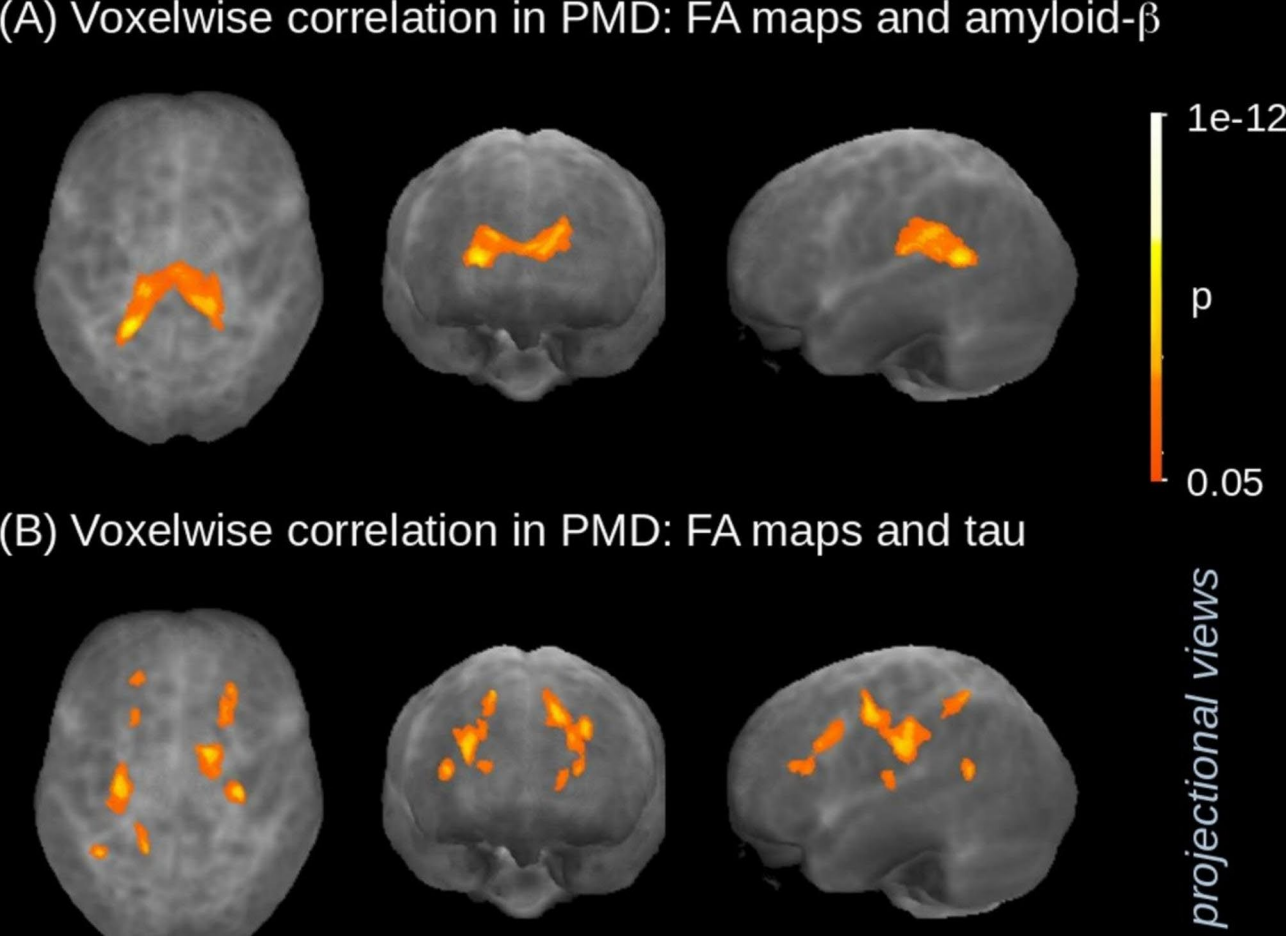
Voxelwise statistical analysis of FA maps of PMS – projectional views. **(A)** Spearman-correlation of FA and amyloid-β shows association to the splenium and the dorsal part of the limbic system. **(B)** Spearman-correlation of FA and tau shows association to the corticospinal tract. Results were thresholded at p < 0.05, corrected for multiple comparisons; cluster threshold > 256 mm³

A significant association cluster for amyloid-ß was observed in the splenium and dorsal part of the cingulum (Fig. 1). For tau protein, significant association clusters were found along the corticospinal tract and along the superior longitudinal fasciculus (Fig. 1; Table 2).

**Table 2:** Association clusters of proteins amyloid-β and tau and FA

No.	MNI (x/y/z)	size / mm³	C / p	anatomical localization
**correlation FA and amyloid-ß**				
1	-22 / -48 / 14	340	0.78 / 0.0001	splenium / posterior part of the corpus callosum
**correlation FA and tau**				
2	-30/-19/29	2420	-0.76 / 0.0002	left corticospinal tract
3	15/-4/46	1663	-0.77 / 0.0001	right frontal lobe
4	24/13/35	1272	-0.76 / 0.0002	right frontal lobe
5	30/-22/37	877	-0.78 / 0.0001	right parietal lobe
6	-17/-51/56	690	-0.72 / 0.0005	left corticospinal tract
7	18/-13/13	533	-0.67 / 0.002	right corticospinal tract
8	-38/-54/18	466	-0.60 / 0.007	left parietal lobe
9	21/14/40	378	-0.68 / 0.001	left frontal lobe
10	22/34/20	294	-0.72 / 0.0005	left frontal lobe

### Results of atlas-based volumetry

The investigation of ICV-corrected GM and WM volumes of PMS patients compared to controls showed significantly reduced WM substructure volumes (p < 0.001, after correction for multiple comparisons), whereas almost no significant differences for GM substructure volumes were observed in the hemispheres (Table 3). The only exception was the putamen, however, this structure is known to show a high variability in ABV determination due to signal inhomogeneities in this structure [12]. Correlations of both the supra- and infratentorial volumetric data (including both WM and GM) with the CSF markers tau protein and amyloid ß did not reveal any significant correlations.

**Table 3:**
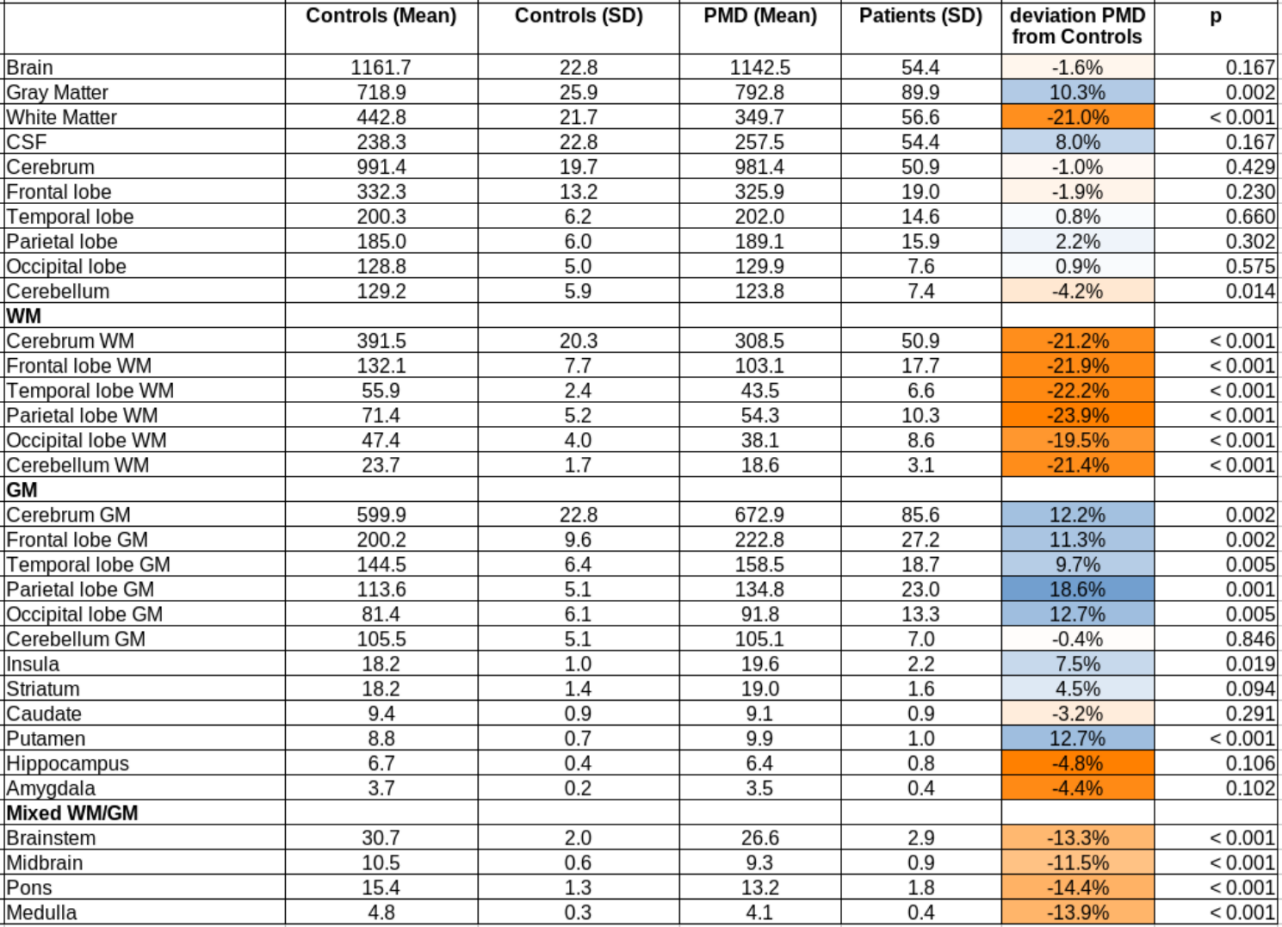
Atlas-based volumetric data. Level of significance was set to p < 0.001 (after correction for multiple comparisons). Abbreviations: CSF = cerebrospinal fluid; WM = white matter; GM = grey matter; SD = standard deviation

## Discussion

PMS is a genetic multisystem disease whose genotype and phenotype have been characterized in increasing detail [13, 14], but whose pathophysiology is only partially understood. Clinical symptoms such as autism spectrum, regression or regressive phases as well as developmental arrest raise the question of whether cerebral inflammatory or degenerative processes play a role in its pathophysiology. To this end, CSF and MRI data from PMS patients were examined. In our PMS cohort, no inflammation in CSF could be demonstrated as neither the basic parameters cell count, lactate, oligoclonal bands nor the additional inflammatory biomarker CXCL13 was pathological. The results were different for markers of neurodegeneration given that the degeneration markers tau protein and amyloid-ß showed pathological values in more than 60% of the patients, regardless of whether there is genetic *SHANK3* involvement or not, according to the current classification into PMS-*SHANK3* related and PMS-*SHANK3* unrelated. However, only descriptive statements can be made here, as the group of PMS-*SHANK3* unrelated with one patient does not allow to make a statistical statement. Alterations were usually detected in just one of these biomarkers. As the typical pattern like in Alzheimer’s disease (i.e., increased tau protein, decreased amyloid-ß, increased p-tau, and decreased amyloid-ß 42/40 ratio) [15] could, thus, not be detected, a typical AD-like neurodegeneration process is apparently not existent in our PMS cohort.

Next, we analysed whether changes in tau and amyloid-ß biomarkers had associations with structural or microstructural measures of the brain, as assessed by MRI. To this end, we observed significant volume reductions in the patients of this study localized in the white matter of supratentorial structures, but not in the grey matter and especially not temporo-parietally or mesiotemporally as one would expect in AD-like pathology. These results led to the assumption that not gross volume changes, but microstructural processes are apparently impaired in PMS patients, as suggested by the very widespread white matter alterations in an unbiased whole brain DTI analysis. Thus, using whole-brain-based spatial statistics of DTI, a voxel-based correlation to tau and amyloid-ß showed a clear pattern for each of the biomarkers: Specifically, the direction of the fibres of the corticospinal tract was negatively correlated with tau values, meaning that pathologically high tau values ​​were associated with a greater reduction in the integrity of the fibres of this motor tract. Possibly, plastic changes in the sense of degenerative and regenerative processes of the microtubules may represent a pathophysiological correlate of these alterations. There is a possible clinical connection between these results and motor functions in the affected patients who have a motor developmental delay with global muscular hypotonia, gait ataxia, and fine motor dysfunction.

The association analysis with amyloid-ß showed a pattern with impairment of the integrity of fibre connections of the splenium and the dorsal cingulum, i.e., the pathologically lower the amyloid-ß values, the lower was the directionality of the nerve fibres. The posterior part of the cingulum receives connections from the posterior parietal cortex, but other cortical afferents to this part of the cingulate gyrus include fibres from the visual and auditory association areas [16]. The functions of these structures relate to memory, in particular working memory, visuo-spatial and autobiographical memory functions [17]. Together with the fibres of the splenium corporis callosi, these fibre tracts are closely connected with the parietal and medial temporal lobes and, according to Broca [18], represent parts of the “large limbic system”. Clinically, patients with PMS show varying degrees of cognitive impairment, ranging from mild intellectual disability to severe cognitive impairment. Learning processes are possible with persistent training and repetition, but deficits in short-term memory often appear, which makes it difficult to learn new memory content. In addition, the patients show affective disorders such as withdrawn behavior as well as attention deficits and impaired adaptation behaviour to environmental stimuli [19] as a further clinical association with the limbic system.

In summary, changes in the neurodegeneration markers tau protein and amyloid-ß in the CSF of patients with PMS seem not to show the typical biomarker pattern of Alzheimer’s pathology. In line with this, there are neither morphological indications of atrophy nor clinically assessable indications of the development of a dementia syndrome. Unexpected was the regional association for both biomarkers with tau/corticospinal tract and amyloid-ß/structures of the limbic system. Based on our microstructural MRI results, one could argue that these results may not reflect degenerative processes sensu stricto, but rather dysfunctional processes which may also be an expression of accelerated aging, as described by Braak and coworkers in autopsies of children and adolescents [20]. Alternatively, the changes could be an expression of neuronal plasticity with constant remodelling and adaptation processes to external stimuli which remains open.

It will be exciting to address these questions with further diagnostic tools like tau/amyloid-PET imaging as well as basic research using iPS cell models and translational *SHANK3* animal models to further characterize the (patho-)physiological assignment of our results.

## Conclusions

More than 60% of the Phelan McDermid patients investigated here have alterations in the CSF degeneration markers tau protein and amyloid-ß without exhibiting the typical pattern seen in patients with Alzheimer disease. The tau and amyloid-ß changes are associated with both functional changes in specific tract systems and clinical symptoms. It remains exciting for further investigations whether these results represent adaptation processes to external stimuli or premature aging processes.

## Materials and methods

### Participants

All patients (mean age 15 ± 16 years, range 2–57 years, 10 males/9 females) had a genetically proven diagnosis of PMS. Genetic diagnoses revealed ten deletions at chromosome 22q13 including *SHANK3* (two of them in the context of an unbalanced translocation with corresponding duplications), one deletion at 22q13.3 without involvement of *SHANK3* (PMS-*SHANK3* unrelated), three cases with disruption of *SHANK3* due to structural abnormalities (ring chromosomes, chromosomal translocation) and five intragenic *SHANK3* variants (Table 4).

**Table 4:** clinical data of PMS patients including age, genetic findings, and stage of development

No	Age	Gender	Genetics	Language	Motor function	ASD	Epilepsy	Cognition	ADHD	Regressive symptoms
1	5	M	arr[GRCh37] 20q13.33(62,908,679)x3, 22q13.33(50915849_51178213)x1	expressive + receptive	Hypotonia	yes	yes	moderate impairment	yes	yes
2	10	F	46, XX, del(22) (q13.3).ish 22q13.3 (*ARSA*-).rev ish dim (22)(q13.3)	expressive	fine motor impairment	yes	no	mild impairment	no	yes
3	22	F	(c.3788dupC; pALA1264Glyfs*32 in *SHANK3*)	expressive + receptive	fine motor impairment	no	no	heavy impairment	no	yes
4	22	M	ring chromosome 22, breakpoint in *SHANK*3	expressive	fine motor impairment	no	no	mild impairment	no	yes
5	56	M	Point mutation in *SHANK*3	expressive	fine motor impairment	no	no	mild impairment	no	no
6	57	M	translocation, breakpoint in *SHANK*3	expressive	fine motor impairment	no	no	mild impairment	no	no
7*	2	F	deletion 22q13.32q13.33, not *SHANK*3-related	expressive + receptive	Hypotonia	yes	no	moderate impairment	no	no
8	3	F	arr[hg19] 22q13.33q13.33(48568738_51219009)x1	expressive + receptive	Hypotonia	yes	no	moderate impairment	no	no
9	29	F	deletion 22q13.33	expressive + receptive	fine motor impairment	yes	no	heavy impairment	no	yes
10	4	M	arr[GRCh37] 22q13.31q13.33 (47922828_51195728)x1	expressive + receptive	Hypotonia	yes	no	heavy impairment	no	no
11	19	F	c.3711_3723; p.Arg1241fs	expressive + receptive	fine motor impairment	yes	no	moderate impairment	no	no
12	15	M	c.2998 G > T; p.Glu1000Ter	expressive + receptive	Hypotonia	no	no	moderate impairment	no	no
13	12	M	microdeletion 22q13.3	expressive + receptive	Hypotonia	yes	no	heavy impairment	no	no
14	4	M	deletion 22q13.33	expressive	Hypotonia	no	no	moderate impairment	no	no
15	4	F	c.3640dupG, p-(Ala1214Glyfs*69)	expressive	fine motor impairment	no	no	mild impairment	yes	no
16	5	M	translocation with microdeletion 22q13, microduplication 1q43q44	expressive + receptive	Hypotonia	yes	no	moderate impairment	yes	no
17	22	F	ring chromosome 22, breakpoint in *SHANK*3	expressive + receptive	fine motor impairment	yes	yes	heavy impairment	no	yes
18	3	M	46 XY, del(22)(q13).ish, del(2q13.1)(*HIRA*+, *SHANK*3-)	expressive + receptive	severe hypotonia	no	no	heavy impairment	no	no
19	4	F	deletion 22q13.33	expressive + receptive	fine motor impairment	yes	no	moderate impairment	yes	no

Abbreviations: ASD = autism spectrum disorder, ASHD = attention deficit hyperactivity disease, * = this patient is PMS-*SHANK3* unrelated

Developmental stages and clinical characteristics of patients were classified for motor skills, speech, cognition, autism and autism spectrum symptoms, attention deficit hyperactivity syndrome, epilepsy, and regressive symptoms (Table 4). Cognition was classified according to the Diagnostic and Statistical Manual of Mental Disorders (DSM-5) [21], autism and autism spectrum by use of the Autism Diagnostic Interview (ADI) and the Autism Diagnostic Observation Schedule (ADOS) [22], while the youngest patients were classified only by clinical impression, and motor as well as speech characteristics were investigated according to clinical diagnostic standards in Germany [23].

### Data acquisition

All subjects or their caregivers gave written informed consent for the study protocol according to institutional guidelines which have been approved by the Ethics Committee of Ulm University, Germany (reference # 321/16) and which were consistent with the declaration of Helsinki.

After informed consent of the patient/caregiver, an anaesthesiologist performed sedation with propofol while continuously monitoring of the vital parameters during MRI acquisition. Directly afterwards, the lumbar puncture was performed while the patient was still sedated. The CSF was immediately taken to the laboratory for further routine diagnostics. The CSF was then frozen at -80 °C, and the biomarkers for the scientific questions were measured at a later point in time.

### CSF diagnostics of biomarkers

Measurement of total-tau (t-tau), pTau181 and Aβ42, Aß40 in CSF: the neurodegenerative biomarkers t-tau, pTau181 and Aβ42, Aß40 in CSF were measured after routine diagnostic work-up of patients. The following assay was used: Fujirebio, Gent, Belgium. Measurement of the inflammatory biomarker CXCL13 was performed using an ELISA (Euroimmun, Lübeck, Germany). Measurement of the axonal biomarker pNF-H was performed using an ELISA (BioVendor, Eching, Germany) (Table 1).

### MRI scanning

MRI scanning was performed on a 1.5 Tesla Magnetom Symphony (Siemens Medical, Erlangen, Germany); the T1-weighted imaging (MPRAGE) consisted of 144 sagittal slices of 1.2 mm thickness, 1.0 mm x 1.0 mm in-plane resolution in a 256 × 248 matrix, echo time (TE) was 4.2 ms, repetition time (TR) was 1640 ms; the diffusion tensor imaging (DTI) study protocol consisted of 52 volumes (64 slices, 128 × 128 pixels, slice thickness 2.8 mm, in-plane pixel size 2.0 mm x 2.0 mm), representing 48 gradient directions (b = 1000 s/mm^2^) and four scans with b = 0, TE and TR were 95 ms and 8000 ms. Further scanning included a T2-weighted data set (Fluid Attenuated Inversion Recovery/FLAIR) with 40 coronal slices of 3.0 mm thickness, 0.45 mm x 0.45 mm in-plane resolution and 512 × 448 voxels matrix dimension, TR/TE 6180ms/112ms).

### DTI data analysis

The postprocessing and statistical analyses were performed by the software platform *Tensor Imaging and Fiber Tracking* (TIFT) [24]. In order to spatially normalize the data to the Montreal Neurological Institute (MNI) stereotaxic standard space, study specific templates were created and MNI normalization was performed iteratively [25]. From the normalized DTI data sets, fractional anisotropy (FA) maps were calculated for quantitative mapping of structural connectivity [26]. In a consecutive step, an 8 mm (FWHM) Gaussian filter was applied for smoothing of FA maps in order to achieve a good balance between sensitivity and specificity. The FA maps of PMS patients and controls were not age-corrected as no correction parameters were available for the 2-year-old PMS patients.

Unbiased WBSS was performed by comparing voxelwise FA values of PMS patients and controls at the group level (non-parametric Mann-Whitney-U-test).

Associations of CSF tau protein and amyloid-ß with FA values were voxelwise calculated by non-parametric Spearman correlation. Both WBSS and the correlation analyses were thresholded at p < 0.05, corrected for multiple comparisons and followed by a clustering procedure discarding isolated clusters (< 256 mm^3^) [27].

### Atlas-based volumetry

As described previously [12, 28], the fully automated method of atlas-based volumetry (ABV) is based on algorithms of SPM (Wellcome Centre for Human Neuroimaging, London, United Kingdom; http://www.fil.ion.ucl.ac.uk/spm) and masks predefined in the reference space by means of different brain atlases. For the purpose of the current study, the volumes of main intracranial compartments as well as gray matter (GM) and white matter (WM) structures were determined according to the LONI Probabilistic Brain Atlas and corrected for intracranial volume (ICV) [12] (Table 3).

### Associations

Associations were calculated by Spearman’s correlation coefficient between the CSF markers tau protein and amyloid-ß and the variables age, gender, genetic findings, language, motor function, ASD, epilepsy, cognition, and ADHD as well as the volumetric results and voxelwise the FA data.

## Data Availability

All data generated or analysed during this study are included in this published article.
